# Knowledge, attitudes, beliefs and behaviour intentions for three bowel management practices in intensive care: effects of a targeted protocol implementation for nursing and medical staff

**DOI:** 10.1186/s12912-015-0056-z

**Published:** 2015-01-31

**Authors:** Serena Knowles, Lawrence T Lam, Elizabeth McInnes, Doug Elliott, Jennifer Hardy, Sandy Middleton

**Affiliations:** School of Nursing, Midwifery and Paramedicine, Australian Catholic University, Australia, and Clinical Nurse Specialist, Intensive Care Service, St. Vincent’s Hospital, Sydney, Australia; Department of Health and Physical Education, The Hong Kong Institute of Education, Hong Kong, Hong Kong; Nursing Research Institute, St Vincent’s Health Australia (Syd) and Australian Catholic University, Sydney, NSW Australia; Faculty of Health, University of Technology, Sydney, Australia; Sydney Nursing School, University of Sydney, Sydney, Australia; Nursing Research Institute, St Vincent’s Health Australia (Syd) and Australian Catholic University, Executive Suite, Level 5, deLacy Building, St. Vincent’s Hospital, 390 Victoria Street, Darlinghurst, NSW Australia

**Keywords:** Bowel management, Intensive care, Nursing, Theory of planned behaviour, Questionnaire

## Abstract

**Background:**

Bowel management protocols have the potential to minimize complications for critically ill patients. Targeted implementation can increase the uptake of protocols by clinicians into practice. The theory of planned behaviour offers a framework in which to investigate clinicians’ intention to perform the behaviour of interest. This study aimed to evaluate the effect of implementing a bowel management protocol on intensive care nursing and medical staffs’ knowledge, attitude, subjective norms, perceived behavioural control, behaviour intentions, role perceptions and past behaviours in relation to three bowel management practices.

**Methods:**

A descriptive before and after survey using a self-administered questionnaire sent to nursing and medical staff working within three intensive care units before and after implementation of our bowel management protocol (pre: May – June 2008; post: Feb – May 2009).

**Results:**

Participants had significantly higher knowledge scores post-implementation of our protocol (pre mean score 17.6; post mean score 19.3; p = 0.004). Post-implementation there was a significant increase in: self-reported past behaviour (pre mean score 5.38; post mean score 7.11; p = 0.002) and subjective norms scores (pre mean score 3.62; post mean score 4.18; p = 0.016) for bowel assessment; and behaviour intention (pre mean score 5.22; post mean score 5.65; p = 0.048) for administration of enema.

**Conclusion:**

This evaluation, informed by the theory of planned behaviour, has provided useful insights into factors that influence clinician intentions to perform evidence-based bowel management practices in intensive care. Addressing factors such as knowledge, attitudes and beliefs can assist in targeting implementation strategies to positively affect clinician behaviour change. Despite an increase in clinicians’ knowledge scores, our implementation strategy did not, however, significantly change clinician behaviour intentions for all three bowel management practices. Further research is required to explore the influence of opinion leaders and organizational culture on clinicians’ behaviour intentions related to bowel management for intensive care patients.

**Electronic supplementary material:**

The online version of this article (doi:10.1186/s12912-015-0056-z) contains supplementary material, which is available to authorized users.

## Background

### Bowel management in intensive care

Maintenance of normal bowel function for a critically ill patient, although often viewed as a low care priority in the highly technical intensive care unit (ICU) environment, is imperative to avoid complications that can delay discharge [1-4]. Critically ill patients are at increased risk of complications from bowel dysfunction due to factors such as reduced mobility, underlying disease process or illness, mechanical ventilation, and the use of continuous or intermittent analgesics [3-5]. Complications include constipation, diarrhoea, delays in mechanical ventilation weaning, greater length of stays, dehydration, and bowel obstruction or perforation [3,6-9].

Protocols can improve bowel management within ICU; guiding clinicians in care provision, ensuring that timely treatment or intervention is instigated, and to minimise complications [1,10-13]. Bowel management protocols (BMPs) have been developed for specific use with ICU patients, with initial evaluations demonstrating a reduction in constipation and diarrhoea [10-15]. Most evaluations of BMP have however only assessed impact on patient outcomes and clinician practices within single site studies; e.g. [15].

Despite the potential for BMPs to standardise care and improve outcomes for critically ill patients, use of protocols is low. Two national surveys in the United Kingdom (UK) found that only 3.5% of ICUs (n = 5) had a guideline for the management of constipation [1], while 21% (n = 17) had a BMP or guideline [14]. In our previous research [16], 32% of 41 responding ICUs in New South Wales, Australia in 2006 had a guideline or protocol for bowel management. This survey also identified bowel management as a practice clinicians viewed as a neglected area [16], similar to findings in the UK [1]. One common limitation with these studies was the lack of detail about the implementation strategies used and the evaluation process.

### Implementation of protocols

Protocols should not be presented to clinicians in isolation, but instead, be introduced with evidence-based implementation strategies to increase their uptake into practice [17,18]. A number of implementation strategies have been described and evaluated in the literature that have demonstrated some effectiveness in changing clinician practices in a variety of settings. These include education, audit and feedback, reminders, mass media, and use of local opinion leaders [19-22]. Central to the process of implementing protocols into clinical practice is clinician behaviour change [23]. Implementation of a protocol requires understanding of what clinicians already do in practice, how the protocol could be adopted within routine practice, and whether clinicians would need to change their practices or behaviours. In addition, behaviour intention is a reliable proxy for actual behaviour when estimating actual clinician practice [24]. Identifying factors that may influence clinician intention to perform behaviours is important for eliciting behaviour change [24,25]. Behaviour intention, the precursor to behaviour performance, is influenced by an individual’s attitudes and beliefs regarding that behaviour [26]. Assessing clinician attitudes and beliefs related to specific behaviours facilitates identification of predictors of behaviour intention and behaviour change.

### Theory of planned behaviour

One model that explains the influences of attitudes and beliefs on behaviour intention is Ajzen’s Theory of Planned Behaviour (TPB) [26]. According to the TPB, an individual’s intention to perform a behaviour can be predicted by determining their attitude toward the behaviour, their beliefs regarding motivation to comply with others expectations (subjective norms) and their beliefs regarding the perceived level of control over factors that may facilitate or hinder their performance of the behaviour (perceived behavioural control). This construct of perceived behavioural control (PBC) can directly influence behaviour, bypassing behaviour intention [26,27]. The control factors of the PBC construct can be either internal or external, with some authors arguing the presence of two distinct constructs or sub-constructs; *self-efficacy* (perceived difficulty); and *controllability* (perceived control) [28,29]. These sub-constructs are seen by some to reflect beliefs about both internal and external factors [30], while others suggest that *self-efficacy* reflects internal factors and *controllability* reflects external factors [28,31]. While the effects of these two PBC sub-constructs have differed across studies, self-efficacy does appear to be a significant positive predictor of behaviour intention [31].

The TPB has been previously used in studies in ICU; to examine the influences of nurse behaviour intention to perform hemodynamic assessment using a pulmonary artery catheter [32], and for changing clinician behaviour with the introduction of care bundles [33]. We undertook a before and after evaluation, not previously done before, of tailored multi-faceted implementation of a BMP into intensive care on clinicians’ knowledge, attitudes, beliefs, role perception and behaviour intentions related to three specific bowel management practices.

## Methods

### Aim

To evaluate the effect of implementing a BMP on the knowledge, attitudes, subjective norms, perceived behavioural control, behaviour intention and role perceptions for ICU nursing and medical staff using three bowel management practices. The following hypotheses were tested:

Nurses and doctors working in the study units post targeted implementation of a BMP, compared to those pre-implementation, would report;

- Higher knowledge scores regarding bowel management practices for intensive care patients
- More positive attitudes towards three bowel management practices
- Greater social pressure to perform three bowel management practices
- Greater perceived behavioural control over performing three bowel management practices
- Greater behaviour intention to perform three bowel management practices
- Higher self-reported past behaviour scores for three bowel management practices
- Greater confidence in deciding when to perform a per rectum examination
- Greater confidence in choosing the correct enema or suppository in relation to per rectum examination results

Clinician perceptions of roles and responsibilities regarding three bowel management practices were also examined.

### Design

A pre and post study was conducted, using self-report, self-administered questionnaires. Data were collected at two time points; pre-implementation and post-implementation of the BMP.

### Participants and recruitment

The study was conducted in three ICUs at a tertiary referral public hospital and a magnet private hospital co-located on the same metropolitan campus, in Australia. Specialties for the three ICUs were cardiothoracic surgery (cardiothoracic ICU), general medical and surgical, including neurology (general ICU) and private, mostly surgical, including cardiothoracic surgery (private ICU).

A list of current nursing and medical staff working in the three ICUs was obtained. Due to staff mobility and rotating rosters it was not possible to follow one sample of staff for the entire study period. Staff who were on extended leave, had resigned or who worked casually were ineligible. Nursing staff with limited direct-care clinical activities, such as nurse unit managers (NUM), clinical nurse educators (CNE), nurse educators (NE), and clinical nurse consultants (CNC) also were excluded. All other nursing and medical staff working in the study units were eligible to participate.

Recruitment of participants for the questionnaires was divided into four phases: pre-notification involving advertisements and advanced letters; round one questionnaire mail out; round two reminder mail out; and round three repeat questionnaire mail out. Sample size calculations were not conducted as the sample was limited to all eligible nurses and doctors employed in the ICUs of the study hospitals.

### Implementation of the new BMP

A BMP was developed by a multidisciplinary team (nurses, doctor, pharmacist, and nutritionist) following review of the literature and existing protocols received from our previous research [16]. A tailored multi-faceted implementation intervention was developed to optimise uptake of the new BMP into practice [22,34]. The implementation intervention consisted of: education sessions, a fact sheet, and reminders, and ran for a period of five months (further details of the BMP and implementation strategy are published in [35]).

### Questionnaire

We developed a questionnaire comprising 98 items divided into six sections; demographics (10 items), knowledge (31 items), three behaviours assessed by TPB constructs (15 items repeated for three behaviour sections), and perceptions of roles and responsibilities (12 items) (Additional file 1).

The knowledge items were guided by previous studies [36-39]; two items used multi-choice response options (one correct answer) while the remaining 29 items had fixed response options of *true, false* or *unsure.* These items assessed knowledge of medications that cause constipation (10 items), medications that cause diarrhoea (10 items) and general bowel management (11 items).

We chose three behaviours to be assessed by the TPB as, they related to bowel management for ICU patients, they were common behaviours ICU clinicians would perform during their roles, and they were specifically detailed in the new BMP implemented as part of this study. The three behaviours were:

1. ***Performing****an assessment of bowel function* (determining presence or absence of: bowel movements, bowel sounds, flatus, distension, tenderness) on an ICU patient at least once every 8 hours (reflected the shift patterns for nurses at the time of the study) for the duration of their ICU admission (herein referred to as ‘*assessment of bowel function*’)
2. ***Performing****a per rectum (PR) examination* on an ICU patient, presented in the context of a scenario of admission day three and bowels not opened since admission (herein referred to as ‘*performing a PR exam*’)
3. ***Prescribing or nurse initiating****the administration of Microlax enema(s)* for ICU patients with a PR exam result of ‘full and soft’ (herein referred to as ‘*administration of enema’*)

We also developed items to measure the constructs of behaviour intention (3 items), attitude (4 items), subjective norm (3 items) and perceived behavioural control (4 items). The four items representing perceived behavioural control were further divided into the sub-constructs of self-efficacy and controllability (2 items each). These items were repeated for the three behaviour sections and scored using a 7-point Likert scale. Past behaviour may influence behaviour intention [40], therefore we included a final item to assess clinicians’ self-reported past behaviour using a response scale of zero to ten.

We complied with Ajzen’s [27] *principle of compatibility* by clearly defining the three behaviours in relation to the elements of Target, Action, Context, and Time (TACT). Vignettes or scenarios assist in defining the intended context of behaviour especially when clinical-related behaviours are complex [41]. We therefore developed scenarios to contextualise the TPB items for two of our behaviours (*performing a PR exam* and *administration of enema*). We used two scenario versions which considered the study ICU specialties; a general patient with sepsis of unknown origin (Gen ICU scenario) and a post-cardiothoracic surgery patient (CT ICU scenario). Scenario versions were allocated to nursing participants based on the study unit in which they worked, while doctors rotated through the study units and consequently scenario versions were randomly allocated.

We designed items to further explore participant perception of roles and responsibilities related to the three behaviours assessed by the TPB [42]. One item assessed participant views on the frequency the behaviour *assessment of bowel function* should be performed and two items assessed participant confidence in *deciding/choosing* related to *performing a PR exam* and *administration of enema* using a 7-point Likert scale. The remaining nine items assessed who were responsible for performing, deciding to perform, and should perform the three behaviours and were presented with eight response options (*the bedside nurse, the nursing team leader, the resident, the registrar, the NUM, the educator, the consultant, other*). An additional response option (*the ICU team (nursing & medical)*) was included in the post-implementation questionnaire, and consequently between group comparisons were not possible for these nine items.

### Questionnaire validity

We determined construct validity of our 14 items designed to measure the TPB constructs and face validity of the scenarios used to contextualize these items for two of the behaviour sections. Briefly, Cronbach’s alpha values were calculated on the pre-implementation responses to determine internal consistency for the TPB construct scales; with ≥ 0.6 considered acceptable [43]. Adequate internal consistencies were achieved for the behaviour intention, attitude and subjective norm constructs for all three behaviours, while the perceived behavioural control construct did not reach adequate internal consistency as a four-item scale for any of the behaviour sections (Table 1). However, a three-item perceived behavioural control construct scale did reach adequate internal consistency for two behaviour sections (*performing a PR exam* and *administration of enema*)*.*

**Table 1:** **Internal consistency for TPB constructs per behaviour**

**Factor**	**Cronbach’s alpha**		
	**Assessment of bowel function (n = 88)**	**Performing a PR exam (n = 88)**	**Administration of enema (n = 88)**
Behaviour Intention (3 items)	0.874	0.926	0.909
Attitude (4 items)	0.839	0.795	0.848
Subjective Norm (3 items)	0.739	0.753	0.773
Perceived behavioural control (4 items)	0.357	0.458	0.578
	0.396 #	0.652 #	0.737 #
Perceived behavioural control: controllability (2 items)	0.370	0.253	0.263
Perceived behavioural control: self-efficacy (2 items)	0.251	0.580	0.722

# if delete ‘factors outside my control’ item.

### Data collection

Data were collected by self-administered questionnaire at two time points; pre-implementation and post-implementation. The pre-implementation survey was conducted from May to July in 2008, directly prior to staff review and implementation of the BMP. The post-implementation survey occurred from February to May 2009, five weeks following the end of the five-month implementation strategy.

### Ethical considerations

Approval to conduct this study was obtained from the Human Research Ethics Committees at St. Vincent’s Hospital (Sydney) and the Australian Catholic University. Participation was voluntary which was explicitly stated in an attached information letter as well as the intention to publish non-identifiable results. By returning the completed survey to the researchers participants gave their implicit consent.

### Data analysis

Data were analysed using SPSS Statistics for Windows, Version 17.0 (SPSS Inc., Released 2008 Chicago, IL, USA). Demographics were described using frequencies. Differences between pre-implementation and post-implementation group responses for independent sample comparisons were examined using t-tests or chi square (*χ*^*2*^*)* procedures. Scores for total knowledge and the three knowledge subsets were calculated for each participant, with frequencies and between-group differences examined. TPB items were recoded to ensure that higher scores correlated with more positive responses and construct scores were calculated by adding responses to the corresponding items and dividing by the number of items in the scale. Descriptive data and between-group differences were examined for individual TPB items and construct scores for each of the behaviour sections. Descriptive statistics were examined for responses to perceptions of roles and responsibilities items.

## Results

### Participants

Of the 130 questionnaires distributed to all relevant staff during the pre-implementation survey (nurses = 103, doctors = 27), 88 (68%) were returned; 76 (86%) from nurses and 12 (14%) from doctors. In the post-implementation survey, 138 questionnaires were distributed (nurses = 110, doctors = 28) and 69 (50%) were returned; 58 (84%) from nurses and 11 (16%) from doctors. Demographic characteristics for both the pre-implementation and the post-implementation data collection points were not significantly different (Table 2).

**Table 2:** **Participant demographics**

**Demographic variable**		**Pre (N = 88)**	**Post (N = 69)**		
		**n (%)**	**n (%)**		**Test statistics**
**Gender**	Female	63(72)	53(77)		x2 = 0.546, *df* = 1, p = 0.46
	Male	25(28)	16(23)		
**Scenario version**	CT ICU scenario	48(55)	45(65)		x2 = 1.824, *df* = 1, p = 0.177
	Gen ICU scenario	40(45)	24(35)		
**Age**	20 - 29	21(24)	21(30)		x2 = 2.566, *df* = 4, p = 0.63
	30 - 39	43(49)	29(42)		
	40 - 49	20(23)	18(26)		
	50 - 59	3(3)	1(1)		
	60 - 69	1(1)			
Current unit*		[n = 76]	[n = 58]		x2 = 3.469, *df* = 2, p = 0.176
	Private ICU	25(33)	17(29)		
	General ICU	32(42)	18(31)		
	Cardiothoracic ICU	19(25)	23(40)		
**Current designation**	RN	56(64)	39(567)		x2 = 5.331, *df* = 6, p = 0.502
	CNS	20(23)	19(27)		
	RMO	3(3)	1(1)		
	Registrar/Senior Registrar	5(6))	7(10)		
	Consultant	4(4)	2(3)		
**Role**	Nurse	76(86)	58(84)		x2 = 0.164, *df* = 1, p = 0.69
	Doctor	12(14)	11(16)		
**Current employment type**	Full Time	64(73)	47(69)		x2 = 1.154, *df* = 3, p = 0.76
	Part Time	22(25)	20(29)		
	Casual/Other	2(2)	1(1)		
**Highest level of education**	Hospital Certificate	3(4)			x2 = 7.35, *df* = 8, p = 0.499
	Associate Diploma/Diploma	8(9)	2(3)		
	Bachelors Degree	39(44)	28(41)		
	Graduate Certificate	21(24)	20(29)		
	Graduate Diploma	6(7)	9(13)		
	Masters Degree	8(9)	8(12)		
	PhD	1(1)	1(1)		
	Other	2(2)	1(1)		
**Enrolled in higher degree study** ^**^**^	Yes	25(29)	13(20)		x2 = 1.642, *df* = 1, p = 0.20
**Level of higher degree study enrolled in** ^**^**^		[n = 25]	[n = 13]		x2 = 3.562, *df* = 4, p = 0.47
	Graduate Certificate/Diploma	14(56)	6(50)		
	Masters Degree by coursework	5(20)	4(33)		
	PhD	2(8)	2(17)		
	Other	4(16)			
	**mean(SD)**	**range**	**mean(SD)**	**range**	
**Years employed in current unit** ^**^**^	5.09(6.09)	3 weeks to 38 yrs	4.61(4.57)	1 month to 18 yrs	t = 0.561, df = 151.63, p = 0.576
**Years of ICU experience**	7.03(6.55)	3 weeks to 38 yrs	6.58(5.70)	1 month to 21 yrs	t = 0.457, df = 153.24, p = 0.649

^^^Missing data; Relevant denominator shown [n = x]; *Only measured for nursing staff.

### Knowledge

Participants’ overall knowledge scores were significantly higher in the post-implementation group when compared to the pre-implementation group (*t = −*2.905, *df =* 153.4, p = 0.004) (Table 3). The post-implementation group scored significantly higher for knowledge of medications that cause diarrhoea (*t = −*2.350, *df =* 148.2, p = 0.02) and knowledge of general bowel management (*t = −*2.499, *df =* 152, p = 0.014) than the pre-implementation group. No significant differences in scores for knowledge of medications that cause constipation were evident (p = 0.23).

**Table 3:** **Bowel management knowledge scores**

	**Pre (n = 88)**		**Post (n = 69)**		
	**Mean (SD)**	**Range**	**Mean (SD)**	**Range**	**Test statistics**
Overall knowledge score (31 items)	17.64 (3.72)	8-25	19.25 (3.22)	11-27	t = −2.905, *df* = 153.43, p = 0.004
Knowledge of medications that may cause diarrhoea (10 items)	4.91 (1.92)	0-9	5.62 (1.86)	1-9	t = −2.35, *df* = 148.154, p = 0.02
Knowledge of general bowel management (11 items)	8.52 (1.49)	5-11	9.09 (1.34)	6-11	t = −2.499, *df* = 152.03, p = 0.014
Knowledge of medications that may cause constipation (10 items)	4.2 (1.61)	2-8	4.54 (1.78)	1-9	t = −1.208, *df* = 138.84, p = 0.229

Maximum possible score for overall knowledge score was 31.

### Behaviour 1: ‘assessment of bowel function’

#### Subjective norm, past behaviour

Participants in the post-implementation group reported higher mean scores for the subjective norm items ‘*My professional colleagues, whose opinion I respect, think that I should perform*’ (*t = −*2.095, *df =* 147.3, p = 0.037); and ‘*I feel under social pressure, from my professional colleagues, to perform*’ (*t = −*2.267, *df =* 139.1, p = 0.02) for assessment of bowel function than those in the pre-implementation group (Table 4).

**Table 4:** **Mean responses to TPB items and construct scores per behaviour (Pre-implementation n = 88; Post-implementation n = 69)**

**TPB constructs**	**TPB items**	**Assessment of bowel function**			**Performing a PR exam**			**Administration of enema**		
		**Mean(SD)**		***p-values***	**Mean(SD)**		***p-values***	**Mean(SD)**		***p-values***
		**Pre**	**Post**		**Pre**	**Post**		**Pre**	**Post**	
Past behaviour	Thinking about the last ten ICU patients you have cared for, for how many of them did you perform^+^	[n = 85] 5.38(3.38)	[n = 63] 7.11(3.22)	0.002	[n = 88] 1.81(2.51)	[n = 64] 2.45(2.98)	0.161	[n = 84] 2.32(3.37)	[n = 58] 2.84(3.72)	0.394
Behaviour Intention^#^	I intend to perform	[n = 88] 5.02(1.86)	[n = 66] 5.45(1.61)	0.125	[n = 86] 4.70(1.86)	[n = 65] 4.75(1.76)	0.850	[n = 88] 5.17(1.56)	[n = 66] 5.64(1.29)	0.044
	I will perform	[n = 88] 5.02(1.83)	[n = 67] 5.25(1.49)	0.388	[n = 85] 5.29(1.75)	[n = 66] 5.26(1.58)	0.893	[n = 87] 5.32(1.5)	[n = 66] 5.65(1.22)	0.136
	I plan to perform	[n = 88] 4.97(1.79)	[n = 65] 5.46(1.48)	0.063	[n = 86] 5.05(1.68)	[n = 66] 5.12(1.67)	0.785	[n = 85] 5.09(1.62)	[n = 65] 5.65(1.27)	0.021
Behaviour Intention (3 item scale)		[n = 88] 5.0(1.63)	[n = 64] 5.41(1.40)	0.101	[n = 85] 5.02(1.65)	[n = 65] 5.03(1.59)	0.970	[n = 85] 5.22(1.43)	[n = 65] 5.65(1.17)	0.048
Attitude^#^	In my opinion, performing X is good practice/bad practice	[n = 87] 6.15(1.30)	[n = 68] 6.09(1.27)	0.768	[n = 83] 5.69(1.34)	[n = 65] 5.55(1.38)	0.557	[n = 84] 5.77(1.29)	[n = 65] 5.78(1.27)	0.959
	In my opinion, performing X is helpful/unhelpful	[n = 81] 5.86(1.47)	[n = 66] 5.83(1.38)	0.896	[n = 79] 5.62(1.34)	[n = 64] 5.52(1.32)	0.641	[n = 80] 5.75(1.29)	[n = 62] 5.71(1.27)	0.852
	In my opinion, performing X is necessary/unnecessary	[n = 82] 5.45(1.78)	[n = 66] 5.62(1.44)	0.522	[n = 83] 5.39(1.61)	[n = 64] 5.22(1.47)	0.515	[n = 81] 5.51(1.42)	[n = 62] 5.69(1.33)	0.417
	In my opinion, performing X is satisfying/unsatisfying	[n = 78] 4.22(1.87)	[n = 65] 4.02(1.88)	0.522	[n = 79] 3.38(1.99)	[n = 64] 3.55(2.01)	0.620	[n = 79] 4.54(1.92)	[n = 62] 4.05(2.16)	0.158
Attitude (4 item scale)		[n = 77] 5.44(1.32)	[n = 65] 5.37(1.28)	0.763	[n = 79] 5.02(1.23)	[n = 63] 4.97(1.3)	0.798	[n = 79] 5.36(1.24)	[n = 62] 5.29(1.26)	0.755
Subjective norms^#^	I feel under social pressure, from my professional colleagues, to perform	[n = 85] 2.39(1.63)	[n = 68] 3.01(1.75)	0.025	[n = 87] 2.67(1.7)	[n = 66] 3.47(1.76)	0.005	[n = 87] 3.31(1.94)	[n = 66] 3.82(1.95)	0.112
	People who are important to me professionally, think that I should perform	[n = 88] 4.32(1.85)	[n = 66] 4.58(1.69)	0.371	[n = 86] 4.44(1.75)	[n = 66] 4.45(1.61)	0.963	[n = 86] 4.43(1.64)	[n = 66] 4.59(1.70)	0.559
	My professional colleagues, whose opinion I respect, think that I should perform	[n = 87] 4.32(1.83)	[n = 66] 4.91(1.62)	0.038	[n = 87] 4.51(1.72)	[n = 66] 4.45(1.66)	0.852	[n = 85] 4.68(1.59)	[n = 65] 4.97(1.50)	0.260
Subjective Norms (3 item scale)		[n = 85] 3.62(1.42)	[n = 66] 4.18(1.36)	0.016	[n = 86] 3.87(1.41)	[n = 66] 4.13(1.37)	0.258	[n = 84] 4.15(1.44)	[n = 65] 4.48(1.46)	0.175
Perceived behavioural control - controllability^#^	I have complete control over performing	[n = 86] 5.38(1.59)	[n = 67] 5.36(1.67)	0.924	[n = 87] 5.15(1.87)	[n = 66] 5.47(1.47)	0.238	[n = 88] 5.26(1.62)	[n = 66] 5.83(1.21)	0.013
	There are factors outside of my control that would prevent me from performing	[n = 88] 3.57(1.84)	[n = 67] 3.64(2.02)	0.816	[n = 85] 3.80(1.93)	[n = 65] 3.57(1.83)	0.455	[n = 85] 3.87(1.86)	[n = 65] 4.05(2.07)	0.591
Perceived behavioural control: controllability (2 item scale)		[n = 86] 4.49(1.35)	[n = 67] 4.5(1.36)	0.979	[n = 85] 4.46(1.44)	[n = 65] 4.51(1.32)	0.829	[n = 85] 4.57(1.33)	[n = 65] 4.93(1.29)	0.091
Perceived behavioural control – self efficacy^#^	I am confident in knowing when an intensive care patient requires	[n = 88] 5.84(1.18)	[n = 67] 5.91(1.11)	0.708	[n = 87] 5.77(1.38)	[n = 66] 5.79(1.20)	0.932	[n = 86] 5.31(1.61)	[n = 65] 5.86(1.18)	0.017
	In my opinion, performing X is very easy/very difficult	[n = 80] 5.31(1.67)	[n = 65] 5.05(1.58)	0.326	[n = 80] 5.20(1.59)	[n = 64] 4.83(1.58)	0.163	[n = 81] 5.58(1.4)	[n = 62] 5.48(1.40)	0.683
Perceived behavioural control: self-efficacy (2 item scale)		[n = 80] 5.54(1.1)	[n = 64] 5.44(1.18)	0.603	[n = 80] 5.46(1.26)	[n = 64] 5.29(1.22)	0.421	[n = 80] 5.42(1.34)	[n = 62] 5.67(1.18)	0.238
Perceived behavioural control (4 item scale)		[n = 80] 5.0(0.92)	[n = 64] 4.91(0.99)	0.578	[n = 80] 4.94(1.05)	[n = 64] 4.88(1.06)	0.758	[n = 79] 4.98(1.08)	[n = 62] 5.26(0.97)	0.102
Perceived behavioural control (3 item scale) #					[n = 80] 5.34(1.25)	[n = 64] 5.33(1.17)	0.967	[n = 80] 5.35(1.25)	[n = 62] 5.71(1.06)	0.067

Relevant denominator shown [n = x]; ^+^Based on a possible range of 0–10 indicating the number of patients for which the behaviour has been performed in the past (self-reported measure); ^#^Based on a possible range of 1–7 with higher scores indicating a more positive response; # if delete ‘factors outside my control’ item.

Those in the post-implementation group reported significantly higher subjective norm construct scores (*t = −*2.434, *df =* 142.8, p = 0.016); and past behaviour scores (*t = −*3.174, *df =* 137.1, p = 0.002) for assessment of bowel function than those in the pre-implementation group (Table 4).

#### Behaviour intention, attitude, perceived behavioural control

There were no statistically significant differences in mean scores for any single item for behaviour intention, attitude or perceived behavioural control between groups (Table 4). There were also no statistically significant differences in the construct scores between groups for behaviour intention (p = 0.1), attitude (p = 0.76) or perceived behavioural control; either as a four item scale (p = 0.58) or split into the two item controllability (p = 0.98) and self-efficacy (p = 0.6) scales (Table 4).

### Behaviour 2: ‘performing a PR exam’

#### Subjective norm

Participants in the post-implementation reported higher mean scores for the subjective norm item ‘*I feel under social pressure, from my professional colleagues, to perform*’ than those in the pre-implementation group (*t = −*2.843, *df =* 137.5, p = 0.005) (Table 4).

#### Behaviour intention, attitude, perceived behavioural control, subjective norm, past behaviour

There was no statistically significant difference in mean scores for any of the behaviour intention, attitude, perceived behavioural control items; and two of the subjective norm items, ‘*People who are important to me professionally, think that I should perform’* and ‘*My professional colleagues, whose opinion I respect, think that I should perform’,* for performing a PR exam between groups (Table 4).

No statistically significant differences were noted in the construct scores for behaviour intention (p = 0.97); attitude (p = 0.8); perceived behavioural control, either as a four item scale (p = 0.76), a 3 item scale (p = 0.97), or split into the two item controllability (p = 0.83) and self-efficacy scales (p = 0.42); subjective norm (p = 0.26); and past behaviour scores (p = 0.16) (Table 4).

### Behaviour 3: ‘administration of enema’

#### Perceived behavioural control, behaviour intention

Participants post-implementation reported higher mean scores for two of the four perceived behavioural control items: ‘*I have complete control over performing*’ (*t = −*2.512, *df =* 152.0, p = 0.013); and ‘*I am confident in knowing when an intensive care patient requires*’ (*t = −*2.407, *df =* 148.9, p = 0.017) for administration of enema than those in the pre-implementation group (Table 4).

Post-implementation participants reported higher mean scores for the behaviour intention items ‘*I plan to perform*’ (*t = −*2.339, *df =* 147.9, p = 0.020); and ‘*I intend to perform*’ (*t = −*2.034, *df =* 150.5, p = 0.044) for administration of enema (Table 4). Participants in the post-implementation also reported significantly higher behaviour intention construct scores for administration of enema than those in the pre-implementation group (*t = −*1.996, *df =* 147.3, p = 0.048) (Table 4).

#### Attitude, subjective norm, perceived behavioural control, past behaviour

There were no statistically significant group differences in mean scores for any of the attitude or subjective norm items; and one of the three behaviour intention items, *‘I will perform’* (Table 4).

For administration of enema, there was no statistically significant difference between groups in the construct scores for attitude (p = 0.75); subjective norm (p = 0.18); perceived behavioural control, either as a four item scale (p = 0.1), a three item scale (p = 0.07) or split into the two item controllability (p = 0.09) and self-efficacy (p = 0.24) scales; and past behaviour scores (p = 0.39) (Table 4).

### Perceptions of roles and responsibilities

Table 5 presents descriptive results for participants’ perceptions of roles and responsibilities for the three behaviours. In both pre-implementation and post-implementation groups the majority of participants indicated in their unit that a nurse performs a bowel function assessment on ICU patients, and that they perceive nurses to have primary responsibility for performing a bowel function assessment.

**Table 5:** **Perceptions of roles and responsibilities and confidence in performing (Pre-implementation n = 88; post-implementation n = 69)**

**Behaviour**	**Item stem**	**Response option group**	**Pre**	**Post**	
			**n (%)**	**n (%)**	**Test statistics**
Bowel assessment	How often should intensive care patients have their bowel function assessed?	Once, on admission		1(1)	
		On admission, and at least once every 8 hours	51(58)	32(46)	
		On day 3 of admission	4(4)	6(9)	
		Other	33(38)	27(39)	
	Who performs bowel assessment	Nurse	66(75)	46(66)	
		Doctor	11(12)		
		ICU Team	N/A	16(23)	
	Who is responsible for bowel assessment	Nurse	71(81)	44(64)	
		Doctor	7(8)	4(6)	
		ICU Team	N/A	16(23)	
PR exam	Who is responsible for PR	Nurse	69(78)	53(77)	
		Doctor	12(14)	4(6)	
		ICU Team	N/A	7(10)	
	Who decides to do a PR	Nurse	46(52)	37(54)	
		Doctor	22(25)	8(12)	
		ICU Team	N/A	18(26)	
	Who should decide to do PR	Nurse	50(57)	39(56)	
		Doctor	17(19)	5(7)	
		ICU Team	N/A	19(28)	
Administration of enema	Who is responsible for administering enema	Nurse	87(99)	62(90)	
		Doctor		1(1)	
		ICU Team	N/A	1(1)	
	Who is responsible for prescribing enema	Nurse	18(20)	19(27)	
		Doctor	53(60)	26(38)	
		ICU Team	N/A	17(27.4)	
	Who is responsible for nurse initiating enema	Nurse	72(82)	55(80)	
		Doctor	4(5)	2(3)	
		ICU Team	N/A	6(9)	
	Who should decide enema	Nurse	36(41)	24(35)	
		Doctor	31(35)	13(19)	
		ICU Team	N/A	27(39)	
**Confidence in performing**			**Mean (SD)**	**Mean (SD)**	
I feel confident in deciding when it is appropriate to perform a PR exam on an intensive care patient^#^			[n = 88] 5.58 (1.68)	[n = 69] 5.79 (1.31)	t = −0.866, *df* = 151.78, p = 0.388
I feel confident in choosing the correct enema or suppository to prescribe/nurse initiate dependant on the results of a PR exam^#^			[n = 88] 4.97 (1.78)	[n = 69] 5.59 (1.34)	t = −2.486, *df* = 152.0, p = 0.014

Where totals do not equal 100%, data were missing; Relevant denominator shown [n = x]; ^#^Based on a possible range of 1–7 with higher scores indicating a more positive response.

Just over half of the participants in the pre-implementation group (n = 51, 58%) indicated a bowel function assessment should be performed *on admission, and at least once every 8 hours* (in line with the new BMP). In contrast, less than half of participants in the post-implementation group (n = 32, 46%) indicated this option, instead responses to *‘other’* included comments that the eight hourly timeframe was not necessary and should be either once or twice per day.

In both the pre-implementation and post-implementation groups just over half of the participants indicated that; within their unit a nurse decides when to perform a PR exam, and that nurses should decide when to perform a PR exam. Over three quarters of participants indicated that in their unit nurses were responsible for performing a PR exam. The majority of participants indicated, that in their unit, it is a nurse who was responsible for administering an enema.

There was a statistically significant difference in the mean scores between groups for responders confidence in choosing the correct enema or suppository dependent on the result of a per rectum examination. Participants in the post-implementation group reported higher mean scores for the item ‘*I feel confident in choosing the correct enema or suppository to prescribe/nurse initiate dependent on the results of a PR exam*’ than those in the pre-implementation group (*t = −*2.486, *df =* 152.0, p = 0.014), thus confirming the hypothesis (Table 5). Following implementation of the BMP, participant confidence in choosing an enema or suppository increased.

There was no statistically significant group difference in mean scores for responders confidence in deciding when to perform a per rectum examination (Table 5). The hypothesis was not confirmed. Confidence in deciding when to perform a per rectum examination was not significantly influenced by implementation of the BMP.

## Discussion

### Key findings

Following implementation of the bowel management protocol, we detected an improvement in clinicians’ overall knowledge scores, knowledge of medications that cause diarrhoea, and knowledge of general bowel management. As education was a key component of our implementation strategy, we expected an improvement in clinicians’ knowledge scores.

We saw a significant increase in the self-reported past behaviour score for behaviour 1: *assessment of bowel function*, indicating that post-implementation clinicians were performing an assessment of bowel function more frequently. Assessment of bowel function is an important aspect of bowel management practices [10]. Assessment was a prominent aspect of our BMP, highlighted in reminders, and was the first element we evaluated to determine clinician compliance with the BMP. However, despite education supporting the importance of frequent assessments of bowel function, responses in the post-implementation group to our item regarding the frequency bowel assessments should be conducted did not support the eight hourly time frame of our BMP, and instead suggested once or twice daily time frames.

Despite also detecting a significant increase in clinicians’ subjective norm scores for assessment of bowel function, we only detected a non-significant increase in behaviour intention during post-implementation of the BMP. Although clinicians in the post-implementation group reported higher past behaviour scores and greater subjective norm scores for bowel assessment, their behaviour intention did not significantly increase. The lack of increase in behaviour intention for assessment of bowel function may be related to the fact that there was no significant change in clinicians’ attitude or perceived behavioural control for this behaviour. It also may be related to participants’ comments indicating that our BMPs requirement for eighth hourly assessment was an unrealistic timeframe.

For behaviour 2: *performing a PR exam*, we only detected a significant change in one of the subjective norm items and not in behaviour intention score or any of the other TPB construct scores*.* Participants’ confidence in deciding when to perform a PR exam did not significantly increase following implementation of our BMP, despite the BMP advocating the performance of a PR exam on day three if a patient had not had their bowels open. It is possible that clinicians are discouraged from intending to perform this behaviour because of the ‘unpleasant’ connotations associated with it [44].

We detected a significant increase in behaviour intention and two PBC items (however, these were not from the same sub-construct) for behaviour 3: *administration of enema.* We also detected a significant increase in responders’ confidence in choosing the correct enema or suppository. Our BMP included an algorithm to guide clinicians in the appropriate action to take dependent on the results of a PR exam, and this may explain clinicians increased intention to prescribe or nurse initiate the administration of an enema for a given PR exam result and their increased confidence in choosing the correct enema or suppository based on the results of a PR exam.

Both behaviours *performing a PR exam* and *administration of enema* were presented in the context of scenarios and required certain criteria to be met before clinicians were required to perform the behaviour. This clear definition of the context for the behaviours is aligned with Ajzen’s [27] principle of compatibility, however, such specificity may have confused clinicians responding to our questionnaire and responses may not be a true indication of clinicians’ intention to perform these behaviours. The lack of a significant change in past behaviour scores for both these behaviours could also be related to there not being a need to perform them for all patients; a PR exam and administration of enema was only advocated if a patients’ bowels had not opened by day three of ICU admission. In comparison, our BMP advocated behaviour 1, *assessment of bowel function,* was performed for all patients. Additionally, the need to perform a PR exam or administer enemas may have been decreased in the post-implementation group if, as our BMP advocated, patients had regular bowel activity as a result of clinicians assessing bowel function and administering aperients. We did not detect any changes in the attitude construct for any of the three behaviours.

We added a response option (the ICU team) in the post-implementation questionnaire for items regarding clinician perceptions of roles and responsibilities. This was in reaction to multiple response options being chosen by participants in the pre-implementation group. We also thought it important to allow this response as one objective of introducing our BMP was for all staff to take responsibility for bowel management and for a ‘team’ management approach to become part of practice. However, comparison between groups was therefore not possible and we also cannot easily determine if responders perceive bowel management to be part of their role.

### Comparison with previous studies

Previous studies investigating nurses’ knowledge of bowel management practices reported an increase in knowledge scores following education sessions [37,38] though neither study was specifically within an ICU setting. However, considered with our other results, an improvement in overall knowledge scores does not necessarily translate into an improvement in clinician behaviour intentions related to bowel management. This highlights the importance of factors other than knowledge in influencing clinician behaviour [45].

Positive attitudes towards guidelines within the ICU have been associated with higher self-reported use of guidelines [46]. The processes clinicians use in making decisions, and not just simply a ‘know-do-gap’, can also influence their use of guidelines [34,47]. Implementation strategies can impact differently on various health care professionals [48,49], however we did not specifically account for differences between clinician groups (nurses and doctors) in our implementation strategy.

We asserted that our targeted implementation strategy would influence clinicians in relation to the TPB constructs of attitude, subjective norms and perceived behavioural control. In particular, by obtaining support from opinion leaders we sought to create greater expectations for clinicians to comply with protocol behaviour from their peers and colleagues, affecting change in social norms [19]. We prompted staff with reminders that were clearly visible to all staff, and that could empower clinicians to act in instigating bowel management for their patients, affecting change in perceived behaviour control [22]. Further, we endeavoured to change attitudes around bowel management by promoting the complications of poor bowel management for critically ill patients in our education sessions and fact sheet.

### Study strengths and weaknesses

Our results showed variability in clinician behaviour intentions and TPB constructs of attitude, subjective norms and perceived behavioural control for three bowel management practices in intensive care following implementation of our BMP. To our knowledge, there have been no previous studies of intensive care clinician bowel management practices utilizing TPB to investigate clinician behaviour intention. Although our study was conducted in 2008–2009, the results remain relevant. There has been little progress in the practice area of bowel management in ICU.

Study limitations are noted. Our study was conducted in three ICUs at two co-located hospitals, and so our sample size was limited to the number of staff working within the units. We were therefore unable to determine differences between nursing and medical staff, given the small response rate from medical staff. Another noted limitation was that we did not include other factors that may influence clinicians’ behaviour intention, such as moral norm [50,51]. We also could have further developed our implementation strategies to specifically address each of the TPB constructs and therefore initiate change in clinician behaviour intentions [52,53]. We acknowledge that behaviour intention and self-reported past behaviour does not necessarily replace objective measures of behaviour [40] and further investigation to determine clinicians actual bowel management practices in intensive care would increase our understandings of this important area. We did not repeat administration of our questionnaire over time. Although sustainability of an intervention is an important issue, this was beyond the scope of our study. Whilst our results were statistically significant, further research is warranted to define parameters to determine clinically meaningful change in clinician behaviour in relation to bowel management.

## Conclusion

Bowel management for critically ill patients is a complex behaviour, and ICU clinicians should consider approaches to ensure their management of bowel function is aligned to minimise complications for patients. Conducting surveys based on the TPB can provide useful insights into factors that influence clinicians’ intentions to perform behaviours and can be used to evaluate the effectiveness of implementing BMPs within ICU. Further refinement of items to measure clinicians’ perceptions of roles and responsibilities regarding bowel management in the intensive care would allow greater insight into their influence on behaviour intention. Ensuring the uptake of BMPs into clinician practice will require further investigation to better understand what influences clinicians’ clinical decisions and behaviours in relation to bowel management. Future investigation into the factors that influence opinion leaders and organizational culture in relation to bowel management may shed light on reasons for the minimal change in clinicians’ behaviour intentions.

## Additional file

Additional file 1:
**Bowel knowledge & TPB survey.**
