# Supplementary material for: Knowledge, attitudes, beliefs and behaviour intentions for three bowel management practices in intensive care: effects of a targeted protocol implementation for nursing and medical staff
Source: BMC Nurs. 2015 Jan 31;14:6. doi: 10.1186/s12912-015-0056-z (PMC4320841; doi:10.1186/s12912-015-0056-z)
Supplement: Additional file 1: — Bowel knowledge & TPB survey. [file 12912_2015_56_MOESM1_ESM.pdf]

SECTION 1 – DEMOGRAPHICS

1. What is your gender? (Please **tick one box only**)

Female .... ☐1
Male .... ☐2

2. What is your age? (Please tick one box only)

< 20 .... ☐1
20-29 .... ☐2
30-39 .... ☐3
40-49 .... ☐4
50-59 .... ☐5
60-69 .... ☐6
≥ 70 .... ☐7

3. What is your current designation? (Please **tick one box only**)

AIN ☐1 EN ☐2 EEN ☐3 RN ☐4 CNS ☐5 CNE ☐6 CNC ☐7 NUM ☐8 RMO ☐9 Reg ☐10
Senior Reg ☐11 Consultant ☐12 Other ☐13
If you chose **'Other'** please specify___________________________

4. How long have you been working in the designation you have selected in question 3? (Please place a number in one of the spaces provided below)

_________ years OR _________ months OR _________ weeks

5. What is your current employment type? (Please **tick one box only**)

Full Time ☐1 Part Time ☐2 Casual ☐3 Other ☐4
If you chose **'Other'** please specify___________________________

6. What is the **highest** level of education you have attained? (Please **tick one box only**)

Hospital Certificate .... ☐1
TAFE Certificate .... ☐2
Associate Diploma .... ☐3
Diploma .... ☐4
Bachelors Degree .... ☐5
Graduate Certificate .... ☐6
Graduate Diploma .... ☐7
Masters Degree .... ☐8
PhD .... ☐9
Other .... ☐10
If you chose **'Other'** please specify___________________________

7. Are you currently enrolled in higher degree study? (Please **tick one box only**)

Yes ☐1 No ☐2

8. If you answered 'yes' to question 7, what is the higher degree you are enrolled in? (Please **tick one box only**)

Graduate Certificate .... ☐1
Graduate Diploma .... ☐2
Masters Degree by coursework .... ☐3
Masters Degree by research .... ☐4
Professional Doctorate .... ☐5
PhD .... ☐6
Other .... ☐7
If you chose **'Other'** please specify___________________________

9. How long have you been employed in this unit? (Please place a number in one of the spaces provided below)

_________ years OR _________ months OR _________ weeks

10. How much experience have you had working in Intensive Care? (Please place a number in one of the spaces provided below)

_________ years OR _________ months OR_________ weeks

SECTION 2

Each question in this section is to test your KNOWLEDGE of Bowel Management practices in Intensive Care. Please tick the appropriate box to indicate TRUE, FALSE or UNSURE.

1. The following medications may cause constipation

a) Morphine .... True ☐1 False ☐2 Unsure ☐3
b) Insulin .... True ☐1 False ☐2 Unsure ☐3
c) Ibuprofen .... True ☐1 False ☐2 Unsure ☐3
d) Iron Supplements .... True ☐1 False ☐2 Unsure ☐3
e) Antiemetics .... True ☐1 False ☐2 Unsure ☐3
f) Antidepressants .... True ☐1 False ☐2 Unsure ☐3
g) Chemotherapy .... True ☐1 False ☐2 Unsure ☐3
h) Calcium Channel Blockers .... True ☐1 False ☐2 Unsure ☐3
i) Anticonvulsants .... True ☐1 False ☐2 Unsure ☐3
j) Antihypertensives .... True ☐1 False ☐2 Unsure ☐3

2. The following medications may cause diarrhoea

a) Morphine .... True ☐1 False ☐2 Unsure ☐3
b) Erythromycin .... True ☐1 False ☐2 Unsure ☐3
c) Ibuprofen .... True ☐1 False ☐2 Unsure ☐3
d) Iron Supplements .... True ☐1 False ☐2 Unsure ☐3
e) Quinidine .... True ☐1 False ☐2 Unsure ☐3
f) Antibiotics .... True ☐1 False ☐2 Unsure ☐3
g) Magnesium .... True ☐1 False ☐2 Unsure ☐3
h) Metoclopramide .... True ☐1 False ☐2 Unsure ☐3
i) Anticonvulsants .... True ☐1 False ☐2 Unsure ☐3
j) Antihypertensives .... True ☐1 False ☐2 Unsure ☐3

3. Daily fluid intake can impact on a patients bowel function .... True ☐1 False ☐2 Unsure ☐3

4. Osmotic laxative medications work by drawing water into the intestine to soften the stool .... True ☐1 False ☐2 Unsure ☐3

5. Patients with decreased mobility are less likely to be constipated .... True ☐1 False ☐2 Unsure ☐3

6. Opiates cause an increase in the propulsive contraction of the gut .... True ☐1 False ☐2 Unsure ☐3

7. A patient may still be constipated even with a bowel movement every day .... True ☐1 False ☐2 Unsure ☐3

8. Stimulant laxative medications work by stimulating the flow of water to the intestine .... True ☐1 False ☐2 Unsure ☐3

9. Difficulty passing stool (straining) may be a sign of constipation .... True ☐1 False ☐2 Unsure ☐3

10. A diagnosis of impaction should be confirmed with radiological investigations such as X-ray .... True ☐1 False ☐2 Unsure ☐3

11. Patients with delayed bowel movements are more likely to have a longer length of stay in the intensive care .... True ☐1 False ☐2 Unsure ☐3

The following questions have multi-choice answer options. Please **tick one box only** indicating the most appropriate answer.

12. Physical assessment of a patients' bowel function includes which of the following?

a) Observation of the abdomen for distension and palpation for tenderness .... ☐1
b) Measuring the head of bed elevation .... ☐2
c) Auscultation for the presence of bowel sounds .... ☐3
d) All of the above .... ☐4
e) a and c .... ☐5

13. For patients with a PR exam result of 'full and hard', the most appropriate initial medication to administer is

a) 1-2 microlax enemas .... ☐1
b) 1-2 glycerine suppositories .... ☐2
c) a phosphate enema (Fleet) .... ☐3
d) All of the above .... ☐4

SECTION 3

Each question in this section refers to **PERFORMING AN ASSESSMENT OF BOWEL FUNCTION FOR INTENSIVE CARE PATIENTS FOR THE DURATION OF THEIR ADMISSION.**

1. Thinking about the last ten intensive care patients that you have cared for, for how many of them did **you** perform an assessment of bowel function at least once every 8 hours for the duration of their admission? ___ / 10

For each of the following questions please circle the number that best matches your point of view.

2. I feel under social pressure, from my professional colleagues, to perform an assessment of bowel function on an intensive care patient at least once every 8 hours for the duration of their admission.

Strongly agree	1	2	3	4	5	6	7	*Strongly disagree*

3. I have complete control over performing an assessment of bowel function on an intensive care patient at least once every 8 hours for the duration of their admission.

Strongly agree	1	2	3	4	5	6	7	*Strongly disagree*

4. I intend to perform an assessment of bowel function on an intensive care patient at least once every 8 hours for the duration of their admission.

Strongly agree	1	2	3	4	5	6	7	*Strongly disagree*

5. In my opinion, performing an assessment of bowel function on an intensive care patient at least once every 8 hours for the duration of their admission is:

Good practice	1	2	3	4	5	6	7	*Bad practice*
Helpful	1	2	3	4	5	6	7	*Unhelpful*
Necessary	1	2	3	4	5	6	7	*Unnecessary*
Satisfying	1	2	3	4	5	6	7	*Not satisfying*
Very easy	1	2	3	4	5	6	7	*Very difficult*

6. I will perform an assessment of bowel function on an intensive care patient at least once every 8 hours for the duration of their admission.

Strongly agree	1	2	3	4	5	6	7	*Strongly disagree*

7. There are factors outside of my control that would prevent me from performing an assessment of bowel function on an intensive care patient at least once every 8 hours for the duration of their admission.

Strongly agree	1	2	3	4	5	6	7	*Strongly disagree*

8. People who are important to me professionally, think that I should perform an assessment of bowel function on an intensive care patient at least once every 8 hours for the duration of their admission.

Strongly agree	1	2	3	4	5	6	7	*Strongly disagree*

9. I plan to perform an assessment of bowel function on an intensive care patient at least once every 8 hours for the duration of their admission.

Strongly agree	1	2	3	4	5	6	7	*Strongly disagree*

10. I am confident in knowing when an intensive care patient requires an assessment of bowel function.

Strongly agree	1	2	3	4	5	6	7	*Strongly disagree*

11. My professional colleagues, whose opinions I respect, think that I should perform an assessment of bowel function on an intensive care patient at least once every 8 hours for the duration of their admission.

Strongly agree	1	2	3	4	5	6	7	*Strongly disagree*

SECTION 4

Each question in this section refers to **PERFORMING A PER RECTUM (PR) EXAMINATION FOR INTENSIVE CARE PATIENTS**. *(delete one scenario)*

Cardiothoracic Case Scenario: Mr Jones was admitted to the Intensive Care Unit following routine cardiothoracic surgery. During his admission, ventilation weaning has been delayed due to poor oxygenation and hemodynamic instability. Mr Jones continues on a morphine infusion at 1-2 mgs per hours for analgesia and an infusion of propofol for sedation. On the day after his admission (Day 1), a nasogastric tube was inserted and enteral feeds were commenced. Mr Jones has been absorbing enteral feeds at a rate of 90 mls per hour with small gastric aspirates. Two coloxyl and senna tablets twice per day were commenced on day 1. Mr Jones has not opened his bowels since admission to the Intensive Care Unit and it is now Day 3. Keeping in mind this scenario, as an example, please complete the following questions.

General Case Scenario: Mr Davies was admitted to the Intensive Care Unit with sepsis of unknown origin. He remains fully ventilated on SIMV and continues on an infusion of morphine at 1-2 mgs per hour and an infusion of propofol for sedation. Enteral feeds were commenced the day after admission (Day 1) at which point two tablets of coloxyl and senna twice per day were also prescribed. He is tolerating the enteral feeds at a rate of 90 mls per hour with small gastric aspirates. Mr Davies has not opened his bowels since admission to the Intensive Care Unit and it is now Day 3. Keeping in mind this scenario, as an example, please complete the following questions.

1. Thinking about the last ten intensive care patients that you have cared for, for how many of them did **you** perform a per rectum (PR) examination? ___ / 10

For each of the following questions please <u>circle</u> the number that best matches your point of view.

2. I feel under social pressure, from my professional colleagues, to perform a per rectum (PR) examination on an intensive care patient.

Strongly agree	1	2	3	4	5	6	7	*Strongly disagree*

3. I have complete control over performing a per rectum (PR) examination on an intensive care patient.

Strongly agree	1	2	3	4	5	6	7	*Strongly disagree*

4. I intend to perform a per rectum (PR) examination on an intensive care patient.

Strongly agree	1	2	3	4	5	6	7	*Strongly disagree*

5. In my opinion, performing a per rectum (PR) examination on an intensive care patient is:

Good practice	1	2	3	4	5	6	7	*Bad practice*
Helpful	1	2	3	4	5	6	7	*Unhelpful*
Necessary	1	2	3	4	5	6	7	*Unnecessary*
Satisfying	1	2	3	4	5	6	7	*Not satisfying*
Very easy	1	2	3	4	5	6	7	*Very difficult*

6. I will perform a per rectum (PR) examination on an intensive care patient.

Strongly agree	1	2	3	4	5	6	7	*Strongly disagree*

7. There are factors outside of my control that would prevent me from performing a per rectum (PR) examination on an intensive care patient.

Strongly agree	1	2	3	4	5	6	7	*Strongly disagree*

8. People who are important to me professionally, think that I should perform a per rectum (PR) examination on an intensive care patient.

Strongly agree	1	2	3	4	5	6	7	*Strongly disagree*

9. I plan to perform a per rectum (PR) examination on an intensive care patient.

Strongly agree	1	2	3	4	5	6	7	*Strongly disagree*

10. I am confident in knowing when an intensive care patient requires a per rectum (PR) examination.

Strongly agree	1	2	3	4	5	6	7	*Strongly disagree*

11. My professional colleagues, whose opinions I respect, think that I should perform a per rectum (PR) examination on an intensive care patient.

Strongly agree	1	2	3	4	5	6	7	*Strongly disagree*

SECTION 5

Each question in this section refers to **PRESCRIBING OR NURSE INITIATING THE ADMINISTRATION OF MICROLAX ENEMA(S) TO AN INTENSIVE CARE PATIENT WITH A PER RECTUM EXAMINATION RESULT OF FULL AND SOFT.** *(delete one scenario)*

Cardiothoracic Scenario: Considering the previous scenario: On performing a per rectum (PR) examination, you discover that Mr Jones' rectum is full and soft. Keeping in mind this scenario, as an example, please complete the following questions.

General Scenario: Considering the previous scenario: On performing a per rectum (PR) examination, you discover that Mr Davies' rectum is full and soft. Keeping in mind this scenario, as an example, please complete the following questions.

1. Thinking about the last ten intensive care patients that you have cared for and that have had a PR examination result of full and soft, for how many of them did **you** prescribe/nurse initiate microlax enema(s)? ______ /10

For each of the following questions please <u>circle</u> the number that best matches your point of view.

2. I feel under social pressure, from my professional colleagues, to prescribe/nurse initiate microlax enema(s) to an intensive care patient who has a PR examination result of full and soft.

Strongly agree	1	2	3	4	5	6	7	***Strongly disagree***

3. I have complete control over whether to prescribe/nurse initiate microlax enema(s) to an intensive care patient who has a PR examination result of full and soft.

Strongly agree	1	2	3	4	5	6	7	***Strongly disagree***

4. I intend to prescribe/nurse initiate microlax enema(s) to an intensive care patient who has a PR examination result of full and soft.

Strongly agree	1	2	3	4	5	6	7	***Strongly disagree***

5. In my opinion, prescribing/nurse initiating microlax enema(s) for an intensive care patient who has a PR examination result of full and soft is:

Good practice	1	2	3	4	5	6	7	***Bad practice***
Helpful	1	2	3	4	5	6	7	***Unhelpful***
Necessary	1	2	3	4	5	6	7	***Unnecessary***
Satisfying	1	2	3	4	5	6	7	***Not satisfying***
Very easy	1	2	3	4	5	6	7	***Very difficult***

6. I will prescribe/nurse initiate microlax enema(s) to an intensive care patient who has a PR examination result of full and soft.

Strongly agree	1	2	3	4	5	6	7	***Strongly disagree***

7. There are factors outside of my control that would prevent me from prescribing/nurse initiating microlax enema(s) to an intensive care patient who has a PR examination result of full and soft.

Strongly agree	1	2	3	4	5	6	7	***Strongly disagree***

8. People who are important to me professionally, think that I should prescribe/nurse initiate microlax enema(s) to an intensive care patient who has a PR examination result of full and soft.

Strongly agree	1	2	3	4	5	6	7	***Strongly disagree***

9. I plan to prescribe/nurse initiate microlax enema(s) to an intensive care patient who has a PR examination result of full and soft.

Strongly agree	1	2	3	4	5	6	7	***Strongly disagree***

10. I am confident in prescribing/nurse initiating microlax enema(s) when an intensive care patient has a PR examination result of full and soft.

Strongly agree	1	2	3	4	5	6	7	***Strongly disagree***

11. My professional colleagues, whose opinions I respect, think that I should prescribe/nurse initiate microlax enema(s) to an intensive care patient who has a PR examination result of full and soft.

Strongly agree	1	2	3	4	5	6	7	***Strongly disagree***

SECTION 6

This section is about **ROLES AND RESPONSIBILITIES** in relation to Bowel Management Practices in Intensive Care and specifically your unit.

For each of the following questions please **tick one box only**.

1. In your view, how often should intensive care patients have their bowel function assessed? **(Tick one box only)**

☐ once, on admission ☐ on admission, and at least once every 8 hours ☐ on day 3 of admission
☐ other (please specify)__________________________________

2. In your unit, who normally performs a bowel function assessment on an intensive care patient? **(Tick one box only)**

☐ the bedside nurse ☐ the nursing team leader ☐ the resident ☐ the registrar ☐ the NUM
☐ the educator ☐ the consultant ☐ the ICU team (nursing & medical)
☐ other(please specify) ______________________

3. In your view, who has primary responsibility for performing a bowel function assessment on an intensive care patient? **(Tick one box only)**

☐ the bedside nurse ☐ the nursing team leader ☐ the resident ☐ the registrar ☐ the NUM
☐ the educator ☐ the consultant ☐ the ICU team (nursing & medical)
☐ other (please specify)______________________

4. In your unit, who normally is responsible for performing a per rectum (PR) examination of a patient? **(Tick one box only)**

☐ the bedside nurse ☐ the nursing team leader ☐ the resident ☐ the registrar ☐ the NUM
☐ the educator ☐ the consultant ☐ the ICU team (nursing & medical)
☐ other (please specify)______________________

5. In your unit, the decision to perform a per rectum (PR) examination of a patient is made by **(Tick one box only)**

☐ the bedside nurse ☐ the nursing team leader ☐ the resident ☐ the registrar ☐ the NUM
☐ the educator ☐ the consultant ☐ the ICU team (nursing & medical)
☐ other (please specify)______________________

6. In your view, who should decide to perform a PR exam on an intensive care patient? **(Tick one box only)**

☐ the bedside nurse ☐ the nursing team leader ☐ the resident ☐ the registrar ☐ the NUM
☐ the educator ☐ the consultant ☐ the ICU team (nursing & medical)
☐ other (please specify)______________________

7. In your unit, who normally is responsible for the **administration** of an enema or suppository? **(Tick one box only)**

☐ the bedside nurse ☐ the nursing team leader ☐ the resident ☐ the registrar ☐ the NUM
☐ the educator ☐ the consultant ☐ the ICU team (nursing & medical)
☐ other (please specify)______________________

8. In your unit, who normally is responsible for **prescribing** an enema or suppository? **(Tick one box only)**

☐ the bedside nurse ☐ the nursing team leader ☐ the resident ☐ the registrar ☐ the NUM
☐ the educator ☐ the consultant ☐ the ICU team (nursing & medical)
☐ other (please specify)______________________

9. In your unit, who normally is responsible for **nurse initiating** an enema or suppository? **(Tick one box only)**

☐ the bedside nurse ☐ the nursing team leader ☐ the resident ☐ the registrar ☐ the NUM
☐ the educator ☐ the consultant ☐ the ICU team (nursing & medical)
☐ other (please specify)______________________

10. In your view, who should decide the appropriate enema or suppository for an intensive care patient? **(Tick one box only)**

☐ the bedside nurse ☐ the nursing team leader ☐ the resident ☐ the registrar ☐ the NUM
☐ the educator ☐ the consultant ☐ the ICU team (nursing & medical)
☐ other (please specify)______________________

PTO

SECTION 6 (Continued)

For the following questions please circle the number that best matches your point of view.

11. I feel confident in deciding when it is appropriate to perform a PR exam on an intensive care patient.

Strongly agree	1	2	3	4	5	6	7	***Strongly disagree***

12. I feel confident in choosing the correct enema or suppository to prescribe/nurse initiate dependant on the results of a PR exam.

Strongly agree	1	2	3	4	5	6	7	***Strongly disagree***
